# Transfusion strategies in bleeding critically ill adults: A clinical practice guideline from the European Society of Intensive Care Medicine: Endorsement by the Scandinavian Society of Anaesthesiology and Intensive Care Medicine

**DOI:** 10.1111/aas.14047

**Published:** 2022-02-28

**Authors:** Morten Hylander Møller, Martin Ingi Sigurðsson, Klaus T. Olkkola, Marius Rehn, Arvi Yli‐Hankala, Michelle S. Chew

**Affiliations:** ^1^ Department of Intensive Care Copenhagen University Hospital Rigshospitalet Copenhagen Denmark; ^2^ Division of Anesthesia and Intensive Care Medicine Landspitali‐The National University Hospital of Iceland Reykjavik Iceland; ^3^ Faculty of Medicine University of Iceland Reykjavik Iceland; ^4^ Department of Anaesthesiology, Intensive Care and Pain Medicine University of Helsinki and Helsinki University Hospital Helsinki Finland; ^5^ Division of Prehospital Services Air Ambulance Department Oslo University Hospital Oslo Norway; ^6^ The Norwegian Air Ambulance Foundation Oslo Norway; ^7^ Faculty of Health Sciences University of Stavanger Stavanger Norway; ^8^ Department of Anaesthesia Tampere University Hospital Tampere Finland; ^9^ Faculty of Medicine and Health Technology Tampere University Tampere Finland; ^10^ Department of Anaesthesia and Intensive Care Biomedical and Clinical Sciences Linköping University Linköping Sweden

**Keywords:** AGREE II, bleeding, clinical practice guideline, critically ill, ICU, transfusion

## Abstract

The Clinical Practice Committee of the Scandinavian Society of Anaesthesiology and Intensive Care Medicine endorses the clinical practice guideline *Transfusion strategies in bleeding critically ill adults*: *a clinical practice guideline from the European Society of Intensive Care Medicine*. This trustworthy clinical practice guideline serves as a useful decision aid for Nordic anaesthesiologists caring for critically ill patients with bleeding.

## 1 BACKGROUND

Critically ill patients, including those in the intensive care unit (ICU) frequently experience bleeding, and this is associated with increased morbidity and mortality.^1^, ^2^

The management of bleeding in critically ill patients is challenging and complex, and often involve multiple concurrent strategies for monitoring coagulopathy, transfusing blood products, and administering medications to support coagulation.^3^

The clinical practice guideline *Transfusion strategies in bleeding critically ill adults*: *a clinical practice guideline from the European Society of Intensive Care Medicine* provides evidence‐based recommendations for transfusion of bleeding in critically ill patients in the ICU.^4^

## 2 METHODS

It was decided by the Clinical practice committee (CPC) of the Scandinavian Society of Anaesthesiology and Intensive Care Medicine (SSAI) to assess the clinical practice guideline *Transfusion strategies in bleeding critically ill adults*: *a clinical practice guideline from the European Society of Intensive Care Medicine* ^4^ for possible endorsement. The Appraisal of Guidelines for REsearch and Evaluation (AGREE) II tool^5^ was used. Details on the endorsement process are available elsewhere.^6^

## 3 RESULTS

**FIGURE 1:**
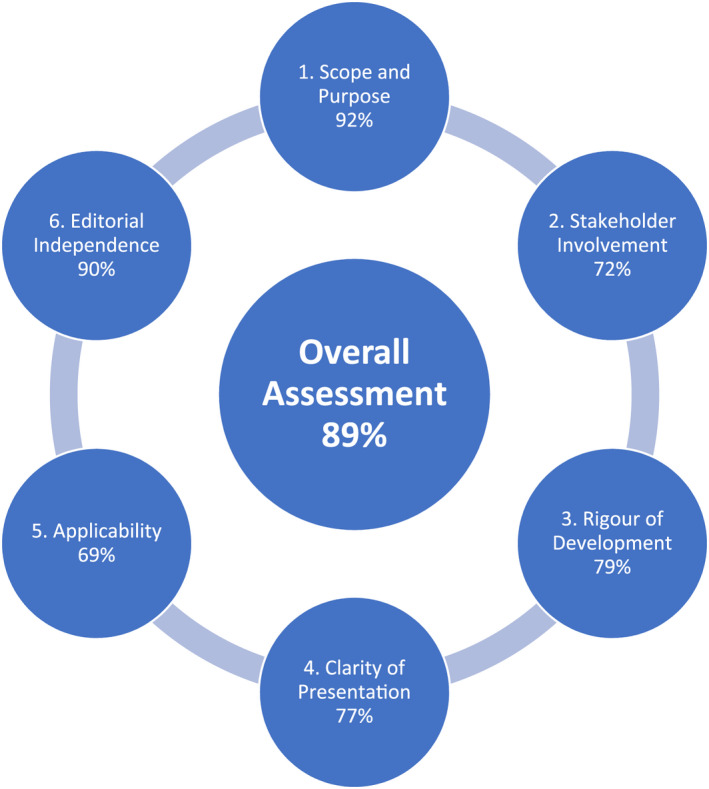
Summary of the appraisal of guidelines for REsearch and Evaluation (AGREE) II assessment^5^

All six SSAI CPC members completed the appraisal. The individual domain totals were: Scope and Purpose 92%; Stakeholder Involvement 72%; Rigour of Development 79%; Clarity of Presentation 77%; Applicability 69%; Editorial Independence 90%; Overall Assessment 89% (Figure 1).

The breakdown of the individual appraisers (de‐identified) is available in the Supporting information.

## 4 DISCUSSION

Agreement between the SSAI CPC appraisers was high, and the overall assessment of the guideline was good. There were minor issues related to stakeholder involvement, as there were no patient representatives on the panel, but patient values and preferences were sought obtained through a literature review. Also, applicability to low resource settings and mass casualties may be limited. Of note, the body of evidence was limited for many questions, why no recommendation was issued in 11 out of 26 questions.

The guideline can be used in daily clinical practice in the Nordic countries without major adaptation or modification.

The clinical practice guideline *Transfusion strategies in bleeding critically ill adults*: *a clinical practice guideline from the European Society of Intensive Care Medicine* ^4^ serves as a useful decision aid for Nordic anaesthesiologists caring for critically ill patients with bleeding.

## 5 CONCLUSION

The SSAI CPC endorses the clinical practice guideline *Transfusion strategies in bleeding critically ill adults*: *a clinical practice guideline from the European Society of Intensive Care Medicine*.^4^

## CONFLICTS OF INTEREST

No Clinical Practice Committee member had direct or indirect conflicts of interest.

## AUTHOR CONTRIBUTIONS

All authors drafted, revised and approved the final manuscript.

## Supporting information

Supplementary Material

## References

[1] Russell L , Holst LB , Kjeldsen L , Stensballe J , Perner A . Risks of bleeding and thrombosis in intensive care unit patients with haematological malignancies. Ann Intensive Care. 2017;7:119. 29230562 10.1186/s13613-017-0341-y PMC5725397

[2] Lauzier F , Arnold DM , Rabbat C , et al. Risk factors and impact of major bleeding in critically ill patients receiving heparin thromboprophylaxis. Intensive Care Med. 2013;39:2135‐2143. 23942857 10.1007/s00134-013-3044-3

[3] Pham HP , Shaz BH . Update on massive transfusion. Br J Anaesth. 2013;111:i71‐i82. 24335401 10.1093/bja/aet376

[4] Vlaar APJ , Dionne JC , de Bruin S , et al. Transfusion strategies in bleeding critically ill adults: a clinical practice guideline from the European Society of Intensive Care Medicine. Intensive Care Med. 2021. doi:10.1007/s00134-021-06531-x PMC8532090 34677620

[5] Brouwers MC , Kho ME , Browman GP , et al.; Consortium ANS . AGREE II: advancing guideline development, reporting and evaluation in health care. J Clin Epidemiol. 2010;63:1308‐1311. 20656455 10.1016/j.jclinepi.2010.07.001

[6] Rehn M , Chew MS , Olkkola KT , Örn Sverrison K , Yli‐Hankala A , Møller MH . Endorsement of clinical practice guidelines by the Scandinavian Society of Anaesthesiology and Intensive Care Medicine. Acta Anaesthesiol Scand. 2019;63:161‐163. 30511469 10.1111/aas.13306

