# Supplementary material for: Transfusion strategies in bleeding critically ill adults: A clinical practice guideline from the European Society of Intensive Care Medicine: Endorsement by the Scandinavian Society of Anaesthesiology and Intensive Care Medicine
Source: Acta Anaesthesiol Scand. 2022 Feb 28;66(5):638–9. doi: 10.1111/aas.14047 (PMC9543689; doi:10.1111/aas.14047)
Supplement: Supplementary file 1 — Supplementary Material [file AAS-66-638-s001.pdf]

A critical group appraisal of: Transfusion strategies in bleeding critically ill adults: a clinical practice guideline from the European Society of Intensive Care Medicine using the AGREE II Instrument

Created with the AGREE II Online Guideline Appraisal Tool.

No endorsement of the content of this document by the AGREE Research Trust should be implied.

Co-ordinator: Morten Hylander Møller

Date: 11 January 2022

Email: mortenhylander@gmail.com

URL of this appraisal: http://www.agreetrust.org/group-appraisal/15740

Guideline URL:
https://link.springer.com/epdf/10.1007/s00134-021-06531-x?sharing_token=4-Ei5sPB6IvfH36b1ECvTfe4RwlQNchNByi7wbcMAY6WQogxXlD_nX6S0nCEVzOmbqNlIOeSryU7nWx4vmkdKj_INssrCVd9vc_ksjmpVl5O05TqPUSf0UgBRrS6uDdTihpbAgRTPJ50PttpyfVGteaLUcGxd1af7Yt9XNr3hec=

Domain 1	Domain 2	Domain 3	Domain 4	Domain 5	Domain 6	OA 1	OA 2
92%	72%	79%	77%	69%	90%	89%	Yes - 6, Yes with modifications - 0, No - 0

Domain 1. Scope and Purpose						
	Appraiser 1	Appraiser 3	Appraiser 7	Appraiser 2	Appraiser 5	Appraiser 4
Item 1	7	6	7	6	7	7
Item 2	7	6	7	6	7	5
Item 3	7	6	7	6	7	6
Domain 2. Stakeholder Involvement						
	Appraiser 1	Appraiser 3	Appraiser 7	Appraiser 2	Appraiser 5	Appraiser 4
Item 4	6	6	7	6	5	6
Item 5	5	3	4	2	1	6
Item 6	7	6	7	6	7	6
Domain 3. Rigour of Development						
	Appraiser 1	Appraiser 3	Appraiser 7	Appraiser 2	Appraiser 5	Appraiser 4
Item 7	7	6	7	7	7	6
Item 8	7	7	7	6	7	6
Item 9	7	6	7	6	7	6
Item 10	7	7	7	6	7	7
Item 11	7	6	7	6	7	7
Item 12	7	6	7	6	7	7
Item 13	7	4	1	1	1	7
Item 14	1	2	2	5	1	4
Domain 4. Clarity of Presentation						
	Appraiser 1	Appraiser 3	Appraiser 7	Appraiser 2	Appraiser 5	Appraiser 4
Item 15	7	5	3	6	5	7
Item 16	7	6	5	6	4	4
Item 17	7	6	7	2	7	7
Domain 5. Applicability						
	Appraiser 1	Appraiser 3	Appraiser 7	Appraiser 2	Appraiser 5	Appraiser 4

Item 18	7	5	7	6	4	5
Item 19	7	5	7	6	5	6
Item 20	7	5	7	6	2	5
Item 21	1	4	6	6	1	4
Domain 6. Editorial Independence						
	Appraiser 1	Appraiser 3	Appraiser 7	Appraiser 2	Appraiser 5	Appraiser 4
Item 22	7	6	7	6	4	6
Item 23	7	7	7	7	7	6
Overall Assessment						
	Appraiser 1	Appraiser 3	Appraiser 7	Appraiser 2	Appraiser 5	Appraiser 4
OA1	7	6	6	6	7	6

Created online at www.agreetrust.org 11 January 2022
